# The impact of China’s low-carbon city pilot policy on public health expenditure

**DOI:** 10.3389/fpubh.2025.1454088

**Published:** 2025-03-21

**Authors:** Yanyin Cui, Jie Ren, Xupeng Gao, Fang Xia

**Affiliations:** ^1^School of Humanities and Management, Zhejiang Chinese Medical University, Hangzhou, Zhejiang, China; ^2^School of Management, Changchun University of Chinese Medicine, Changchun, Jilin, China

**Keywords:** low-carbon city pilot policy, public health expenditure, public low-carbon behavior, air pollution, time-varying DID simulation

## Abstract

**Objective:**

In driving a transition in environmental governance, China’s low-carbon city pilot (LCCP) policy has exerted a dichotomous impact on public health expenditure that is characterized by both a decline in relative proportion and expansion of absolute scale. Research to date is insufficient for understanding the transmission mechanisms and policy coordination pathways underlying this contradiction, which has hindered the sustainable realization of environmental health benefits. This study thus investigates the impact of China’s LCCP policy on public health expenditure and the underlying mechanisms involved.

**Methods:**

Based on panel data of 285 Chinese cities at the prefecture level and above from 2003 to 2019, a quasi-natural experiment was conducted using China’s LCCP policy. The time-varying difference-in-differences simulation method and hierarchical regression method were used to analyze the effect and mechanism of the LCCP policy on China’s public health expenditure.

**Results:**

The results demonstrate the inherently paradoxical nature of the effects of the LCCP policy on public health expenditure: although the LCCP policy produces a significant relative reduction in public health expenditure (*β* = −0.331, *p* < 0.001), it simultaneously produces a pronounced expansion in terms of absolute expenditure (*β* = 0.409, *p* < 0.001). These impacts are spatially heterogeneous across regions and exhibit supply–demand divergence in healthcare infrastructure readiness and environmental threshold effects that are contingent upon pollution severity gradients. Further analysis of the underlying mechanism reveals that public low-carbon behaviors serve as dual negative mediators in both expenditure dimensions, whereas household medical burdens exert a significant positive mediating effect on absolute expenditure but a statistically insignificant mediating effect on relative expenditure.

**Conclusion:**

This study reveals the complex synergistic mechanisms linking environmental governance to health investment allocation. The internal contradictory effects of the LCCP policy on public health expenditures must be resolved by striking a balance between environmental governance and health investment, implementing regional differentiation strategies, optimizing the structure of preventive expenditures, and guiding the public to collaborative participation. China’s environmental quality and public health should be promoted simultaneously.

## 1 Introduction

Worldwide, nations have been struggling to reconcile their decarbonization targets with their socioeconomic development priorities, as evidenced by the continuing and persistent growth of carbon emissions despite multilateral climate commitments (1, 2). This tension is acutely manifested in China’s green low-carbon transition, in that plans for rapid emissions reductions coincide with plans to expand public health investments, creating a paradoxical synergy that demands systematic investigation. With the comprehensive promotion of China’s green low-carbon economic transformation and the goal to build a healthy nation, three batches of national low-carbon provincial and municipal pilots were launched by China’s National Development and Reform Commission in 2010, 2012, and 2017 in 81 cities and six provinces (3). The Environmental Kuznets Curve has been objectively shown to apply to China, and China’s requirements for economic development in the short term have intensified environmental pressure there (4). Presently, expanding production at the cost of environmental quality can drive economic development, but doing so will harm the physical health of residents, increase the demand for medical and health services, and objectively increase the financial burden of livelihood-related expenditures. Low-carbon cities are cities with low-carbon economies; in these cities, economic income increases despite controlled carbon emissions. The aim of this initiative is to improve public health and achieve strong coordination among the individual parts of the “economy-environment-health” system. With the development of China’s low-carbon economy and the ongoing social progress, the people have increasingly made strong demands for a healthy ecological environment and improved medical conditions. These demands affect public health expenditure (5, 6).

The quality of public health expenditure tends to be reflected by public health outcomes. Most related studies have noted that positive public health outcomes are obtained through an increase in public health expenditure (7, 8). Typically, long-term exposure of an area to environmental pollution causes the deterioration of the health of residents, which leads to notable increases in public health spending (9). Pollution is not exogenous but rather, it is related to both the level and pattern of local economic development. When endogenous factors and an exogenous environment change this process, the government’s public health system reform directly reflects the diversity of public health expenditure (10). Governments often curb environmental pollution through environmental regulation policies—measures that will certainly affect public health expenditure. Previous literature has confirmed the following: (1) Environmental pollution stimulates governments to increase public health expenditures, and (2) a relaxation of environmental regulations promotes regional economic development in the short term. However, a deteriorating ecological environment will also increase the burden of government health expenditure (11).

Safeguarding and enhancing the livelihoods of individuals within a development framework constitutes a key objective of Chinese-style modernization. Public health expenditures and environmental quality represent two critical areas related to livelihood-related expenditures. Most existing studies pertaining to these issues focus on two primary categories: The first category of the literature predominantly examines health outcomes associated with environmental regulatory policies. This category integrates the impacts of such regulations on pollution control and explores the interconnections between environmental enhancement and public health from the perspective of environmental health economics. Through the lens of the triadic dynamic relationship of “policy-environment-health,” environmental regulatory policies have been found to exert a beneficial influence on the prevention of environmental pollution, thereby promoting public health (12). Research has demonstrated that China’s environmental regulatory policies substantially influence public health by enhancing air quality management, which in turn lowers healthcare expenditures (13). Furthermore, these regulations encourage the adoption of various sustainable and health-promoting practices, including the transition to clean energy (14), waste separation (15), and low-carbon transportation (16, 17). These practices improve environmental quality, thereby contributing to public health and further decreasing healthcare costs. It has been projected that the 13th Five-Year Plan for Ecological and Environmental Protection will decrease national healthcare expenditure by $47.36 billion annually, representing 0.64% of China’s gross domestic product (18).

The second category of the literature examines welfare and economic outcomes associated with environmental regulatory policies. This body of research examined the influence of pertinent environmental regulations on enhancing ecological welfare performance from an ecological welfare perspective. Furthermore, the impact of environmental regulation on ecological wellbeing was analyzed in terms of its effects on both environmental quality and human wellbeing. The relationship between economic development and the enhancement of livelihoods is characterized by mutual reinforcement rather than mutual exclusivity. From the perspectives of the “forcing effect” and “innovation compensation,” enhancing environmental standards compels polluting enterprises to undertake technological upgrades, thereby advancing industrial development toward increased sophistication (19, 20). Most studies concur the existence of a significant interaction between health expenditure and economic growth; in this interaction, economic growth is essential to facilitate increased levels of government health spending. Environmental regulations yield economic advantages that encourage fiscal investment in public health, which, in turn, serves as an investment in healthy human capital, thereby contributing to enhanced regional development (21). Consequently, environmental policy can ideally achieve a triple dividend, encompassing sustained economic growth, improved environmental quality, and an increased scale of public health spending.

In conclusion, China’s low-carbon city pilot (LCCP) policy has an asymmetric effect on public health expenditure, and policymakers should avoid being misled by the illusion of absolute quantity. Environmental regulation enhances public health by improving environmental quality, which consequently leads to a certain degree of reduction in public health expenditure. Concurrently, the economic advantages derived from environmental regulation encourage local governments to prioritize the wellbeing of their residents, potentially leading to increased investment in public health infrastructure. Research to date has predominantly examined the health effects of the LCCP policy through unidimensional analytical frameworks, either emphasizing synergistic benefits from pollution abatement that reduces disease burdens or critiquing conflict risks by which environmental investments displace social welfare resources. These methodologies have left the structural ramifications of low-carbon transition on public health expenditure architecture largely underexplored, with empirical investigations into the dualistic dynamics of expenditure (i.e., simultaneous contraction and expansion effects) remaining at a nascent stage of theoretical development. In China, a contemporary paradox has emerged that prevents a deeper understanding of the implications and demands of novel concepts in the current phase of development. This paradox necessitates a balance between the advantages and disadvantages of environmental quality, public health needs, and economic development. Based on existing research, in this paper, a quasi-natural experiment is designed, using panel data of Chinese cities from 2003 to 2019. The data are associated with China’s low-carbon pilot city policies and are used to study their impact on public health expenditure. (1) The theoretical mechanism of the effect of the LCCP policy on public health expenditure is identified. (2) The impact of the LCCP policy on both absolute public health expenditure and relative public health expenditure is assessed using a time-varying difference-in-differences (DID) simulation, and robustness tests are conducted. (3) Sub-sample regression is used to explore heterogeneities in the impact of the LCCP policy on public health expenditure in terms of regions, medical infrastructure, and pollution levels. (4) The internal mechanisms of the influence of the LCCP policy on public health expenditure through the mediators of public low-carbon behaviors and the medical expenditure burden on residential households are explored.

The marginal contributions of this paper are as follows: Firstly, the growth of absolute public health expenditure relies on the expansion of the total fiscal volume, while the decline in relative public health expenditure exposes a priority shift in the allocation of fiscal resources. This contradiction indicates that the compatibility of environmental governance with health equity is highly dependent on institutional design. This paper introduces the indicators of relative and absolute public health expenditure, reveals the asymmetric effect of low-carbon policies on public health, and provides a new perspective for interdisciplinary research on fiscal issues related to the environment and health. Secondly, by constructing a dual-index (i.e., relative vs. absolute) evaluation framework, this study reveals the mechanisms by which the policy constraints and environmental health benefits of the LCCP policy affect public health expenditures. The aim is to provide a new theoretical explanation, analytical framework, and literature supplement for the causal relationship linking environmental regulation to public health expenditures. Thirdly, given that public health and environmental protection are pivotal priorities for enhancing the quality of life, this paper delves into the interrelationship between these domains by examining whether they are complementary or mutually exclusive. The findings offer theoretical insights and serve as a decision-making reference to advance environmental welfare for residents within the context of the new development paradigm. Thereby, this paper contributes to the realization of Chinese modernization in harmony with nature.

## 2 Theoretical hypotheses

**Figure 1:**
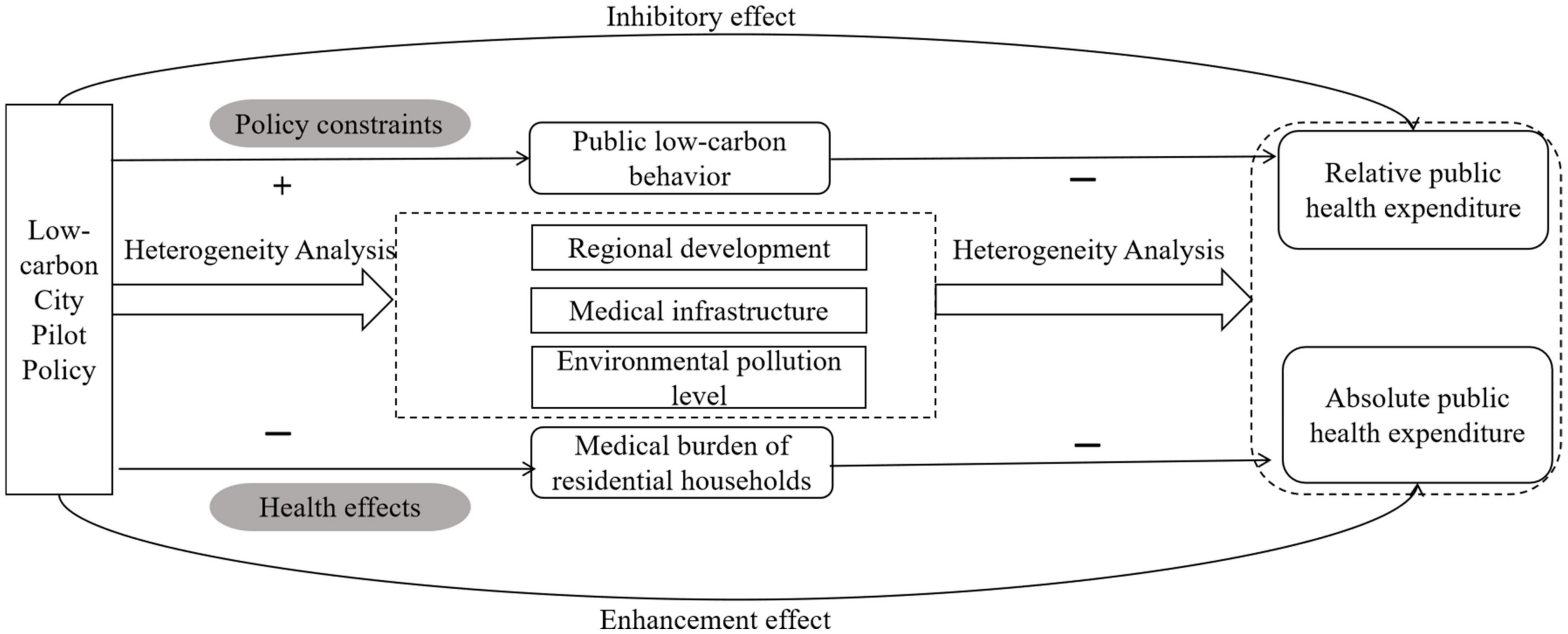
Diagram of the transmission mechanism.

This paper discusses the effect of China’s LCCP policy on public health expenditure from the perspectives of both the direct mechanism and indirect mechanism (Figure 1). The direct mechanism explores the direct impact of the LCCP policy on public health expenditure; the indirect mechanism explores the intrinsic mechanism of the LCCP policy on public health expenditure in terms of both public low-carbon behavior and medical burden on residential households.

### 2.1 Direct impact of China’s LCCP policy on public health expenditures

As a comprehensive environmental regulation measure, China’s LCCP program requires the joint participation of multiple entities for its implementation. The national government has a high degree of autonomy, which has a compound impact on public health expenditure. The LCCP policy mainly affects public health expenditure through fiscal resource redistribution and the lagged effect of health improvement (22–24).

In terms of the redistribution of financial resources, the LCCP policy requires the local governments of pilot cities to invest a large amount of funds in green infrastructure, technological research and development, and pollution control. This leads to a shift in the structure of fiscal expenditure toward environmental protection, but this squeezes public health expenditure as a relative proportion of total fiscal expenditure (25). At the same time, the LCCP policy also requires local governments of pilot cities to conduct rigid assessments of environmental protection targets, thereby driving local governments to carefully weigh the relationship between the use of energy resources and economic growth in their respective regions (26). Environmental regulation serves as a fundamental component of environmental governance and provides an institutional assurance for economic development. It represents a crucial strategy for achieving the dual harmonization of environmental quality and high-quality economic growth. The relationship between environmental regulation and high economic quality exhibits a U-shaped pattern. Once the degree of environmental regulation surpasses the inflection point of this pattern, further intensification of regulations can significantly enhance the development of high economic quality (27). Low-carbon policies can drive economic growth or attract special central fiscal transfer payments, thereby increasing the government’s total disposable fiscal revenue and supporting, in turn, the growth of the absolute amount of public health expenditure. As low carbon cities develop, public health expenditure—an important subordinate allocation of government expenditure—will increase in scale.

In terms of the lagged effect of health improvement, most studies to date have suggested that environmental regulation is an effective way to improve public health. Chay et al. (28), Luechinger (29), Greenstone et al. (30), Tanaka et al. (31), and Wang et al. (30) have studied environmental regulations such as the Clean Air Act in the United States, the desulfurization policy of power plants in Germany, the catalytic converter policy in India, and the Two Control Zone policy in China, respectively. They found that, in general, environmental regulation can improve respiratory health status and reduce the population mortality rate. In the short term, the LCCP policy directly reduces the incidence rate of respiratory diseases by reducing pollution. It thus reduces therapeutic expenditures in the public health system and thereby slows the growth rate of public health expenditures as a relative proportion of the total. In the long run, this improvement of environmental quality may reduce the burden of chronic diseases. The government may use the funds it thereby saves to expand preventive public health services, resulting in an increase in the absolute amount of public health expenditures.

In general, the LCCP policy has led to a simultaneous relative decrease in public health expenditure and an absolute increase in public health expenditure. In the short term, it may limit the availability of resources in other areas related to people’s livelihood, but in the long term, it can contribute to fiscal sustainability through the improvement of public health. Based on this, this study proposes the following hypothesis regarding the dual paths of the expenditure of low-carbon pilot cities on public health expenditure:

*H1:* The LCCP policy reduces the relative amount of public health expenditure but expands absolute public health investment.

### 2.2 Indirect effects of China’s LCCP policy on public health expenditures

The changes in public health expenditure allocation mechanisms are not solely dependent on the government’s subjective decisions; they also result from the combined effects of public participation and health dividends. The fundamental cause of the dual-path conflict in public health expenditure under the LCCP policy stems from the government dominating the adjustment of expenditure structures without considering the reverse effect of public pressure on fiscal demands. The key to addressing this contradiction lies in designing buffer mechanisms from the public perspective. This section examines the indirect impacts of low-carbon environmental policies by analyzing both the constraints they impose on public behavior and their health benefit outcomes.

#### 2.2.1 Policy constraints: the mediating effect of the public’s low-carbon behaviors

Following a low carbon lifestyle is a significant predictor of residents’ perceptions of health co-benefits (32). The LCCP policy exemplifies the government’s commitment to addressing environmental pollution by implementing urban low-carbon development planning, systems, and policies. Concurrently, this policy serves to disseminate information regarding environmental protection and the outcomes of urban environmental governance for both social organizations and individuals, thereby augmenting the environmental awareness of the people (33). The LCCP policy, through infrastructure improvement, economic incentives, and publicity and education, has led to a shift of financial resources toward the environmental protection sector, resulting in a decrease in public health expenditure as a relative proportion of the whole (34). China’s LCCP policy has contributed to the formation of low-carbon living concepts and increased participation in low-carbon behaviors. In turn, this has significantly reduced the carbon emission intensity of residents’ lives and improved their green living standards (35). The notion of low-carbon environmental protection has catalyzed the robust growth of green industries, including prefabricated construction, eco-friendly furniture, and sustainable transportation; thereby, the development of urban life characterized by green and low-carbon principles has been enhanced (36, 37). Green urban living can effectively improve the health level of residents and thus relatively reduce the demand for health services. When low-carbon behaviors become widespread, the marginal cost of environmental protection expenditures decreases, thereby alleviating the financial burden of public health expenditures (30). The following hypothesis is thus proposed:

*H2:* China’s LCCP policy eases the financial burden of public health expenditure by promoting low-carbon public behaviors.

#### 2.2.2 Health dividend: the mediating effect of the medical burden on residential households

The implementation of the LCCP policy presents an optimistic picture to the public regarding the prospective enhancement of environmental quality. This perception can subtly influence both health demand and real income through the health effect and the pass-through effect, consequently affecting residents’ healthcare expenditures. Relevant studies have demonstrated that environmental regulations compel firms to substantially reduce their pollutant emissions. Conversely, environmental regulations may directly diminish the after-tax revenues of firms, potentially impacting their payroll levels and subsequently reducing workers’ healthcare expenditures (38, 39). Consequently, a decline in the healthcare spending of people results in a corresponding decrease in the proportion of public health expenditure funded by financial subsidies. As environmental quality enhances, the depreciation rate of individual health capital diminishes. This leads to a reduction in health damage attributable to environmental pollution, thereby decreasing the healthcare expenditures of the people (40). The improvement of health directly reduces residents’ medical expenses. This decrease in households’ average annual out-of-pocket medical expenses will slow the growth of expenditures from the medical insurance fund (41). It is worth noting that the surplus of medical insurance has not been automatically converted into investment in public health. Local governments tend to instead invest surplus funds in projects that can showcase obvious political achievements, resulting in a decline in the proportion of public health expenditure out of the total financial expenditure. Based on this, this study proposes as follows:

*H3:* The LCCP policy alleviates the financial burden of public health expenditure by reducing the medical expenses on residential households.

## 3 Methods and data

### 3.1 Methods

#### 3.1.1 Time-varying DID simulation method

To examine the impact of the LCCP policy on public health expenditure, this paper uses the introduction of this policy as a quasi-natural experiment. In this experiment, low-carbon pilot cities are the experimental group, and non-pilot cities are the control group. Given that China announced three batches of low carbon pilot cities in 2010, 2012, and 2017, the following time-varying DID simulation method is constructed:


(1) $$y_{\mathrm{it}}\left(yR_{\mathrm{it}},yA_{\mathrm{it}}\right)=\alpha_{1}+\beta_{1}did_{\mathrm{it}}+\gamma X_{\mathrm{it}}+v_{t}+v_{c}+\varepsilon_{\mathrm{it}}$$

where, *i* is the sample city, *t* is the year, and the dependent variable *y_it_* refers to the public health expenditure of city *i* in year *t* (*yR_it_* is the relative public health expenditure and *yA_it_* is the absolute public health expenditure); *did_it_* represents the LCCP policy, *X_it_* refers to a series of control variables, *v_t_* is the time fixed effect, and *v_c_* is the individual city fixed effect; *ε_it_* is the random error term, *α_1_* is the constant term, *γ* is the coefficient of the control variable, and *β*_1_ refers to the net effect of the LCCP policy. This paper focuses on both the positive and negative aspects of the coefficient *β*_1_.

#### 3.1.2 Hierarchical regression method

The LPPC policy may impact public health expenditure through public low-carbon behavior and environmental pollution. Therefore, a mediating effect test model and a moderating test model are constructed, both based on hierarchical regression. The aim is to confirm the intrinsic mechanism. The following mediating effect model is constructed:


(2) $$y_{\mathrm{it}}\left(yR_{\mathrm{it}},\;yA_{\mathrm{it}}\right)=\alpha_{1}+\beta_{1}did_{\mathrm{it}}+\gamma X_{\mathrm{it}}+v_{t}+v_{c}+\varepsilon_{\mathrm{it}}$$

(3) $$M_{\mathrm{it}}=\alpha_{2}+\beta_{2}did_{\mathrm{it}}+\gamma X_{\mathrm{it}}+v_{t}+v_{c}+\varepsilon_{\mathrm{it}}$$

(4) $$y_{\mathrm{it}}\left(yR_{\mathrm{it}},yA_{\mathrm{it}}\right)=\alpha_{3}+\beta_{3}did_{\mathrm{it}}+\beta_{4}M_{\mathrm{it}}+\gamma X_{\mathrm{it}}+v_{t}+v_{c}+\varepsilon_{\mathrm{it}}$$

where *M_it_* refers to the mediating variable of public low-carbon behavior, and the remaining variables have the same meaning as in Equation 1. Firstly, the significance of *β_1_* is tested in Equation 2. If *β_1_* is significant, the analysis proceeds to the next step; if it is not significant, the analysis stops. Secondly, the significance of *β_2_* is tested in Equation 3. If *β_2_* is significant, this means that there is a mediating effect for the subsequent test. Thirdly, whether *β_3_* and *β_4_* in Equation 4 are significant is assessed. If both are significant, this means that there is a partial mediating effect of the mediating variable.

The moderating effect model is constructed through a “two-step approach,” as follows:


(5) $$y_{\mathrm{it}}\left(yR_{\mathrm{it}},yA_{\mathrm{it}}\right)=\alpha_{1}+\beta_{1}did_{\mathrm{it}}+\beta_{5}W_{\mathrm{it}}+\gamma X_{\mathrm{it}}+v_{t}+v_{c}+\varepsilon_{\mathrm{it}}$$

(6) $$\begin{array}{c} y_{\mathrm{it}}\left(yR_{\mathrm{it}},yA_{\mathrm{it}}\right)=\alpha_{1}+\beta_{1}did_{\mathrm{it}}+\beta_{5}W_{\mathrm{it}}+\beta_{6}W_{\mathrm{it}}\\ \times DID_{\mathrm{it}}+\gamma X_{\mathrm{it}}+v_{t}+v_{c}+\varepsilon_{\mathrm{it}} \end{array}$$

where *W_it_* refers to the moderating variable of air pollution, *W_it_* × *DID_it_* is the product of the independent variable (DID) and the moderating variable (air pollution), and the remaining variables have the same meaning as above. Firstly, the moderating variable is added to the baseline model Equation 1 to arrive at Equation 5. If *β_1_* and *β_5_* are significant, the interaction term between independent variables and moderating variables can continue to be added, thus arriving at Equation 6. If the interaction term coefficient *β_6_* is significant, *W_it_* plays a moderating role.

### 3.2 Variables

#### 3.2.1 Explained variables

Fiscal health expenditure is used as an agent variable for absolute public health expenditure. This variable indicates the absolute magnitude of investment in public health services over a specific period of time. The share of health care expenditure in fiscal expenditures—a relative public health expenditure variable—measures the relationship between the public resources consumed for health care services and the public resources consumed by other public utilities over a defined period of time. Because of a lack of data on prefecture-level cities, this paper uses data of the province where the prefecture-level city is located as a proxy variable for prefecture-level cities.

#### 3.2.2 Key explanatory variables

The DID variable is the product of the low carbon city dummy (to which a value of 1 is assigned for low carbon pilot cities, and 0 otherwise) and the pilot time dummy (to which a value of 1 is assigned for the year of policy implementation, and 0 otherwise). In this paper, cities that were established as national low carbon pilots in 2010, 2012, and 2017 are selected as the intervention group; other cities are selected as the control group. The first batch of pilot low-carbon cities was launched on July 19, 2010, the second batch on November 26, 2012, and the third batch on January 7, 2017. Therefore, 2010, 2013, and 2017 are defined as policy starting years of the three batches of pilot low-carbon cities.

#### 3.2.3 Mediating variables

Electricity consumption behaviors exhibit both direct and indirect linkages with carbon emissions. Low-carbon electricity consumption by residents is China’s first carbon-inclusive mechanism to focus on residents’ daily electricity conservation behavior. This study quantifies public low-carbon behaviors by applying the maximum value subtraction method to positively normalize the urban household electricity consumption data. Household medical burden is measured by healthcare expenditures as a share of total household consumption; this indicator comprehensively incorporates family members’ medical needs and the cost of medical services.

#### 3.2.4 Control variables

The DID model based on panel data itself has good robustness and has been well controlled for endogeneity as well as both time and individual fixed effects. To further reduce the estimation bias caused by other omitted variables, this study also selected variables that affect the experimental results other than the experimental factors for use as control variables. Specifically, based on the examples of prior studies (21, 42, 43), variables such as medical infrastructure construction, economic development, pollution control, financial freedom, and urban people’s livelihood construction were introduced as control variables. These control variables were introduced for the following reasons. Healthcare infrastructure controls were included for the confounding effects of regional resource endowment disparities. Economic development variables disentangle the confounding effects between economic scale and policy effectiveness. Pollution control capacity identifies synergistic/offsetting effects between low-carbon policies and environmental governance. Fiscal autonomy accounts for heterogeneity in local government financial behaviors. Urban livelihood development captures competitive dynamics in multidimensional public expenditure allocations. The specific variables are detailed in Table 1.

**Table 1:** Descriptive statistics of variables.

Category	Variable	Variable name	Variable definitions	Sample size	Average value	Standard deviation
Explained variables	*Re_phex*	Relative public health expenditure	Share of health care expenditure in financial expenditure	4,845	0.066	0.020
	*Ab-phex*	Absolute public health expenditure	Natural logarithm of financial health care spending	4,845	5.905	0.756
Key explanatory variables	*DID*	DID	Interaction term of low carbon city dummy variables with pilot time dummy variables	4,845	0.202	0.402
Adjustment variables	*Air_Pol*	Air pollution	PM2.5 concentration (μg/m^3^)	4,845	42.644	18.918
Mediator variables	*LC_beh*	Public low-carbon behavior	Natural logarithm of urban residential electricity consumption	4,845	10.809	1.187
	*MB_rh*	Medical burden of residential households	The proportion of medical and health care consumption in resident households (%)	4,845	7.301	1.635
Control variables	*Eco_dev*	Economic development	Natural logarithm of per capita gross regional product	4,845	10.516	0.807
			Natural logarithm of the actual amount of foreign investment used	4,845	8.546	2.724
	*He_inf*	Healthcare infrastructure	Number of practicing or assistant physicians (persons per 10,000 people)	4,845	38.517	18.428
			Number of medical institutions (units per 10,000 people)	4,845	0.621	0 0.761
			Number of hospital beds (beds per 10,000 people)	4,845	56.760	32.667
	*Pol_ab*	Pollution control	Harmless treatment rate of domestic waste (%)	4,845	82.916	25.408
			Centralized sewage treatment rate (%)	4,842	74.244	139.893
			Treatment rate of industrial solid waste (%)	4,845	77.942	27.205
	*LI_con*	Livelihood construction	Number of buses per 10,000 people (vehicles per 10,000 people)	4,845	7.619	7.122
			Share of education spending in fiscal spending	4,845	0.165	0.059
			Green coverage rate of the built-up area (%)	4,844	37.271	13.411
	*Fis_aut*	Fiscal autonomy	Fiscal spending as a percentage of GDP	4,845	0.165	0.196

The GDP is acronym for the gross domestic product.

### 3.3 Data sources

In this study, data on 285 Chinese cities at the prefecture-level and above from 2003 to 2019, were selected as study sample. Because of severe data deficiencies or administrative division adjustments during the sample period, cities such as Chaohu, Sansha, Haidong, Danzhou, and Laiwu were excluded from the sample. PM_2.5_ concentration data were obtained from the Atmospheric Composition Analysis Group database. Public health expenditure data were obtained from various China Statistical Yearbooks and China Health Statistical Yearbooks. Other city-related data were obtained from the China City Statistical Yearbook and each city’s government gazette of previous years. Because of the difficulty associated with obtaining the data of prefecture-level cities, certain missing data points were supplemented according to the average growth rate. The variables of total financial health care expenditure, per capita gross regional product, urban residents’ domestic electricity consumption, and actual use of foreign investment are logarithmically processed (using the natural logarithm). This was done to eliminate the influence of the quantitative scale.

## 4 Results

### 4.1 Time-varying DID baseline regression result

Table 2 presents the baseline regression results for the effect of China’s LCCP policy on absolute public health expenditure, as compared to public health expenditure. Columns (1)–(6) sequentially display the results of gradually adding control variables on the basis of controlling for time effects and city effects. The coefficients of the policy dummy variable for relative public health expenditure are all significantly negative at the 1% confidence level, while the coefficients for absolute public health expenditure are all significantly positive at the 1% confidence level. Changes in the control variables did not affect the significance of these policy effects, and the regression results are very robust. Hypothesis 1 is thus supported.

**Table 2:** Time-varying regression results for China’s LCCP policy and public health expenditure.

Variable	Relative public health expenditure						Absolute public health expenditure					
	Model (1)	Model (2)	Model (3)	Model (4)	Model (5)	Model (6)	Model (1)	Model (2)	Model (3)	Model (4)	Model (5)	Model (6)
*DID*	−0.336*** (0.08)	−0.325*** (0.08)	−0.323*** (0.08)	−0.320*** (0.08)	−0.331*** (0.08)	−0.331*** (0.08)	0.423** (0.17)	0.420*** (0.16)	0.426*** (0.15)	0.429*** (0.15)	0.414*** (0.15)	0.409*** (0.15)
Economic development		Yes	Yes	Yes	Yes	Yes		Yes	Yes	Yes	Yes	Yes
Healthcare infrastructure			Yes	Yes	Yes	Yes			Yes	Yes	Yes	Yes
Pollution control				Yes	Yes	Yes				Yes	Yes	Yes
Livelihood construction					Yes	Yes					Yes	Yes
Fiscal autonomy						Yes						Yes
Urban fixed effects	Yes	Yes	Yes	Yes	Yes	Yes	Yes	Yes	Yes	Yes	Yes	Yes
Time fixed effects	Yes	Yes	Yes	Yes	Yes	Yes	Yes	Yes	Yes	Yes	Yes	Yes
*Cons*	4.246*** (0.04)	2.648*** (0.80)	2.646*** (0.82)	2.606*** (0.80)	2.210*** (0.83)	2.208*** (0.83)	45.595*** (0.07)	43.785*** (1.36)	43.863*** (1.30)	43.890*** (1.30)	43.607*** (1.26)	43.619*** (1.25)
*N*	4,845	4,845	4,845	4,842	4,841	4,836	4,845	4,845	4,845	4,842	4,841	4,836
*R-squared*	0.884	0.885	0.886	0.886	0.887	0.887	0.976	0.976	0.976	0.976	0.976	0.976

Standard Error in parentheses, ***, **, and * mean passing significance test at 1, 5, and 10% confidence levels, respectively.

The results of the benchmark regression indicate that the LCCP policy had a significant effect of reducing the relative amount of public health expenditure, while increasing the absolute amount of public health expenditure. With no control variables added, the coefficient of the effect of reducing the relative amount of public health expenditure was −0.336. After adding the control variables, the reduction effect weakened to −0.331. Meanwhile, in the absence of control variables, the effect of promoting the absolute amount of public health expenditure was 0.423. After adding the control variables, the promotion effect decreased to 0.409. This shows that in regions with well-developed medical infrastructure, the structure of health expenditure has become stable, resulting in a stronger adaptability to the LCCP policy that reduces its effects. In economically developed regions, the relative proportion of health expenditure can be maintained by expanding the scale of expenditure, thus weakening the effect of the LCCP policy. There may be a policy substitution effect involving pollution control and the low-carbon pilot policy, which could amplify the synergistic expenditure reduction effect of the LCCP policy. In addition, regions with a high degree of financial autonomy can flexibly allocate resources, and this, too, can weaken the impact of the LCCP policy on expenditure. The squeezing of the health budget by livelihood projects may exacerbate the reduction in public health expenditure resulting from the LCCP policy.

### 4.2 Robustness test

#### 4.2.1 Parallel trend hypothesis testing

**Figure 2:**
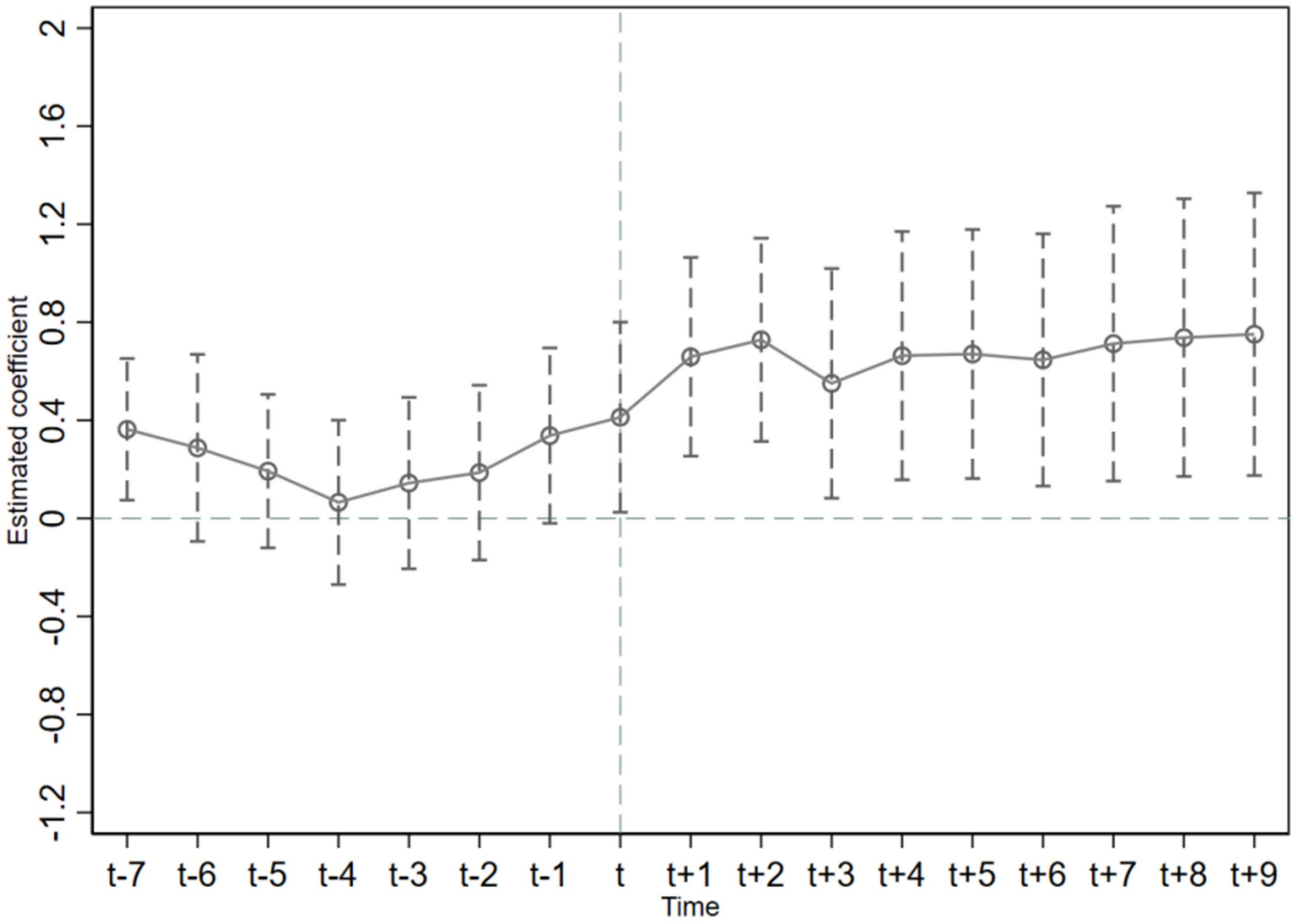
A parallel trend test of the *Re-phex.*

**Figure 3:**
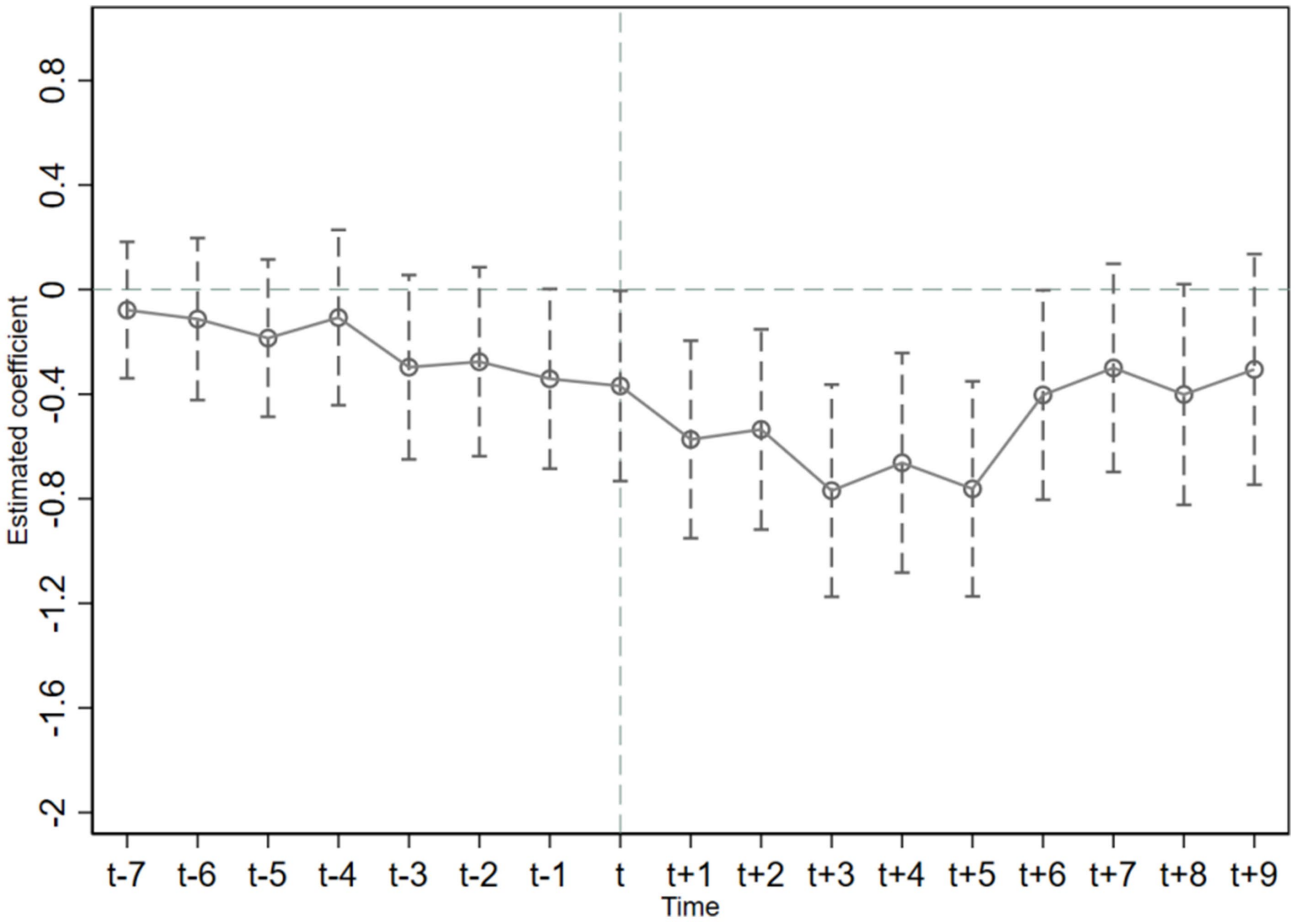
A parallel trend test of the *Ab-phex.*

The premise for using the time-varying DID method to evaluate the effect of policy implementation is to satisfy the hypothesis of parallelism. That is, before the implementation of the policy, the trends of public health expenditure in low-carbon pilot cities and other cities should be similar. Figures 2, 3 show that the estimated DID coefficients were not significant prior to the implementation of the policy; coefficients fluctuated around 0. This finding indicates that prior to the implementation of the LCCP policy, no significant difference in the variation trend of public health expenditure existed between treatment group and control group. This conforms to the parallel trend hypothesis. As shown in Figure 2, the estimated DID coefficient in the 5 years after the implementation of China’s LCCP policy was significant and decreased year by year. In the sixth year, the estimated coefficient again began to fluctuate around 0. This finding shows that the LCCP has an increasing reduction effect on relative public health expenditure in the short term, and this reduction effect is not apparent from the sixth year onwards. In Figure 3, the estimated DID coefficient was not significant in the current period and was only significant after *t* + 1. This finding indicates that the improvement of absolute public health expenditure induced by the LPPC policy has a time lag effect of about 1 year.

In conclusion, from the dynamic effect, the reduction effect of the LCCP policy on relative public health expenditure is a short-term effect. Moreover, the promotion effect on absolute public health expenditure has a time lag and long-term dimension.

#### 4.2.2 Test of the difference-in-differences method with propensity score matching (PSM-DID)

The LCCP policy is, in essence, a non-randomized (i.e., quasi-natural) experiment. The selection of pilot cities in China for the LCCP may be affected by their initial endowments, and thus the selection is not completely random. Drawing on the research of Shipman (44), this study uses the nearest neighbor matching method with a caliper of 0.01 and the smallest nearest neighbor to conduct a 1:1 non-repeating nearest neighbor matching. After matching, the deviation of almost all variables between the treatment group and the control group is controlled within a reasonable range, indicating good matching quality. After propensity score matching, PSM-DID can be further used for regression analysis (see Table 3). Although the regression results of the two explained variables are numerically different from the previous benchmark regression results, the effect is consistent with those results. This shows that after eliminating the self-selection bias of the experimental group, the regression results are still robust.

**Table 3:** Results of the robustness test.

Variable	PSM-DID		Addressing competing explanations		Controlling for contemporaneous policy confounding	
	*Re_phex*	*Ab-phex*	*Re_phex*	*Ab-phex*	*Re_phex*	*Ab-phex*
*DID*	−0.254** (0.10)	0.534*** (0.11)	−0.290*** (0.07)	0.431** (0.16)	−0.331*** (0.08)	0.409*** (0.15)
Control variables	Yes	Yes	Yes	Yes	Yes	Yes
Urban fixed effects	Yes	Yes	Yes	Yes	Yes	Yes
Time fixed effects	Yes	Yes	Yes	Yes	Yes	Yes
Cons	10.497*** (1.05)	63.622*** (1.30)	2.461*** (0.81)	43.921*** (1.21)	−1.538** (0.69)	25.501*** (1.08)
*N*	4,005	4,005	4,513	4,513	4,836	4,836
R-squared	0.928	0.991	0.893	0.978	0.887	0.976

Standard Error in parentheses, ***, **, and * mean passing significance test at 1, 5, and 10% confidence levels, respectively.

#### 4.2.3 Addressing competing explanations

There are significant differences between, on the one hand, sub-provincial-level cities and cities at higher administrative levels, and, on the other, more ordinary cities in terms of economic scale, policy resources, and administrative status, making it difficult to take conclusions about the effects of the policy on the former class of cities and generalize them to ordinary cities. In this study, regression analysis was conducted after excluding extreme samples (i.e., sub-provincial-level cities and cities at higher administrative levels) to eliminate the estimation bias caused by differences in administrative levels and economic status, and to verify the universality of the effects of the LCCP policy on ordinary cities. The results (see Table 3) show that after reducing the sample of pilot cities, the regression remains highly significant, and the sign of the coefficient is consistent with that of the baseline regression. This indicates that the conclusion is robust and not unduly influenced by the inclusion of special cities.

#### 4.2.4 Controlling for contemporaneous policy confounding

In October 2016, the Central Committee of the Communist Party of China and the State Council issued the outline of the Healthy China 2030 Plan, which clearly places health in a strategic position of priority development. This health policy could impact the effect of the LCCP policy on public health expenditure and thus had to be taken into account in our analysis. After adding the dummy variable of this policy in the regression analysis, the regression coefficients of the two explained variables are still significant at the 1% level, and the estimation results remain robust and reliable (see Table 3).

### 4.3 Heterogeneous analysis of the impact of LCCP policy on public health expenditure

#### 4.3.1 Regional heterogeneity

Regional development in China is unbalanced, and thus the division of China into the eastern, central, and western regions can effectively reflect the systematic gradient differences among these regions in terms of economic structure, fiscal capacity, environmental foundation, and health needs. These differences in the resource endowments and economic development of different cities imply that the promulgation and implementation of low-carbon policies will also produce different policy effects. Ignoring this spatial differentiation will lead to either overestimation or underestimation of policy effects, potentially misleading policymakers. Table 4 reports the results of the analysis of regional heterogeneity in the impact of the low-carbon city pilot policy on public health expenditure. The effect of the LCCP policy in reducing relative public expenditure was only significant in the eastern region (*β* = −0.312, *p* < 0.001), but not significant in the central and western regions (*β* = −0.059, *p* > 0.1; *β* = −0.211, *p* > 0.1). Meanwhile, the effect of the LCCP in increasing absolute public expenditure was significant in the central region (*β* = 0.642, *p* < 0.001), was not significant in the eastern region (*β* = 0.264, *p* > 0.1), and showed a significant effect of decreasing absolute public expenditure in the western region (*β* = −0.229, *p* < 0.1). These results confirm the effects of regional heterogeneity and indicate that differences in the effects of the low-carbon city pilot policy stem from differences in resource endowments and development stages among regions.

**Table 4:** Results of the regional heterogeneity analysis.

Variable	Relative public health expenditure			Absolute public health expenditure		
	Eastern Region	Central Region	Western Region	Eastern Region	Central Region	Western Region
*DID*	−0.312*** (0.11)	−0.059 (0.11)	−0.211 (0.15)	0.264 (0.22)	0.642*** (0.20)	−0.229* (0.12)
Control variables	Yes	Yes	Yes	Yes	Yes	Yes
Urban fixed effects	Yes	Yes	Yes	Yes	Yes	Yes
Time fixed effects	Yes	Yes	Yes	Yes	Yes	Yes
Cons	4.931*** (1.09)	3.624*** (0.786)	−0.014 (2.04)	42.943*** (2.28)	42.474*** (1.74)	43.443*** (1.05)
*N*	1,699	2,088	1,049	1,699	2,088	1,049
R-squared	0.881	0.923	0.903	0.972	0.980	0.993

Standard Error in parentheses, ***, **, and * mean passing significance test at 1, 5, and 10% confidence levels, respectively.

#### 4.3.2 Healthcare infrastructure heterogeneity

Exploring the effects of heterogeneity of medical infrastructure on the impact of the LCCP policy on public health expenditure reveals the differentiation of policy effects from the supply side. In 2023, six departments, including the National Health Commission of China, announced the selection of 81 pilot cities for the construction of close-knit urban medical groups. The selection of these pilot cities prioritized consideration of each city’s pre-existing medical resource base. Therefore, the selection of a city as a pilot city for the construction of a close-knit urban medical group was used here as a proxy for the level of local medical infrastructure. The analysis results show (see Table 5) that the inhibitory effect of the LCCP policy on relative public health expenditure was significant in general areas (*β* = −0.407, *p* < 0.001), but not significant in areas with abundant basic medical resources (*β* = −0.091 *p* > 0.1). The effect and significance of the LCCP policy in increasing absolute public health expenditure were both higher in areas with abundant medical basic resources (*β* = 0.531, *p* < 0.05) than in general areas (*β* = 0.334, *p* < 0.1). This indicates that the impact of the LCCP policy on public health expenditure is deeply dependent on regional medical resource endowment.

**Table 5:** Results of the heterogeneity analysis in the medical infrastructure.

Variable	Relative public health expenditure		Absolute public health expenditure	
	Areas with abundant basic medical resources	General areas	Areas with abundant basic medical resources	General areas
*DID*	−0.091 (0.15)	−0.407*** (0.09)	0.531** (0.24)	0.334* (0.19)
Control variables	Yes	Yes	Yes	Yes
Urban fixed effects	Yes	Yes	Yes	Yes
Time fixed effects	Yes	Yes	Yes	Yes
*Cons*	−0.286 (0.895)	2.812*** (0.69)	38.929*** (2.47)	38.929*** (0.69)
*N*	1,205	3,631	1,205	3,631
*R-squared*	0.871	0.899	0.977	0.977

Standard Error in parentheses, ***, **, and * mean passing significance test at 1, 5, and 10% confidence levels, respectively.

#### 4.3.3 Heterogeneity in the pollution grade regions

There is a nonlinear relationship between pollution levels and health needs, and thus regional heterogeneity in pollution levels was examined here to explore the effect of the LCCP policy on public health expenditure from the demand side. According to the annual historical data from the *China Ecological and Environmental Status Bulletin* and the government’s pollution control documents, the pollution levels in the various regions of China are categorized into high, medium, and low pollution regions. The high-pollution regions include the Beijing-Tianjin-Hebei region and its surrounding areas, the Fenhe-Weihe Plain, and some cities in Xinjiang; the medium-pollution regions include the urban agglomeration in the middle reaches of the Yangtze River, the Chengdu-Chongqing region, and the Northeast Industrial Belt; and the low-pollution regions include the southeastern coastal areas, the southwestern ecological barrier region, the Qinghai-Tibet Plateau, and Hainan. The analysis results (see Table 6) indicate that the LCCP policy had a significant inhibitory effect on relative public health expenditures in the different pollution regions. However, the reduction in the low-pollution region was the highest (*β* = −0.609, *p* < 0.01), followed by the high-pollution region (*β* = −0.473, *p* < 0.01), with the medium-pollution region being the lowest (*β* = −0.220, *p* < 0.05). The LCCP policy only had a significant effect of increasing the absolute amount of public health expenditure in the low-pollution region (*β* = 0.694, *p* < 0.01), and had a significant reduction effect in the high-pollution (*β* = −0.494, *p* < 0.01) and medium-pollution regions (*β* = −0.051, *p* < 0.01). Of the latter two, the reduction effect in the high-pollution region was significantly higher than that in the medium-pollution region.

**Table 6:** Results of the regional heterogeneity analysis.

Variable	Relative public health expenditure			Absolute public health expenditure		
	Highly polluted area	Moderately polluted area	Lowly polluted area	Highly polluted area	Moderately polluted area	Lowly polluted area
*DID*	−0.473*** (0.21)	−0.220** (0.10)	−0.609*** (0.19)	−0.494*** (0.08)	−0.051*** (0.80)	0.694*** (0.19)
Control variables	Yes	Yes	Yes	Yes	Yes	Yes
Urban fixed effects	Yes	Yes	Yes	Yes	Yes	Yes
Time fixed effects	Yes	Yes	Yes	Yes	Yes	Yes
*Cons*	4.331*** (1.13)	0.666 (1.24)	4.212 (2.83)	45.135*** (0.80)	40.708*** (2.29)	51.686*** (2.28)
*N*	1,258	2,565	1,013	1,258	2,565	1,013
*R-squared*	0.921	0.885	0.918	0.994	0.974	0.989

Standard Error in parentheses, ***, **, and * mean passing significance test at 1, 5, and 10% confidence levels, respectively.

### 4.4 Analysis of the impact mechanism of China’s LCCP policy on public health expenditure

#### 4.4.1 Testing the mediating mechanism of public low-carbon behavior

Table 7 presents the results of the test of the mediating mechanism of the public’s low-carbon behavior. The regression coefficient of *DID* for the public’s low-carbon behavior is 0.081 and is significant at the 5% confidence level, indicating that the LCCP policy promotes the public’s environmental protection awareness and low-carbon behavior. Once the mediating variables are included, the coefficient of *DID* for relative public health expenditure is 0.321 and is significant at the 1% level, while the coefficient for the public’s low-carbon behavior is −0.119 and is significant at the 5% confidence level. Meanwhile, the coefficient of *DID* for absolute public health expenditure is 0.435 and is significant at the 1% level, while the coefficient of the public’s low-carbon behavior is −0.318 and is significant at the 1% confidence level. This shows that the LCCP policy significantly promotes the public’s low-carbon behavior, which in turn reduces both the relative and absolute amounts of public health expenditure. Hypothesis 2 is thus supported.

**Table 7:** Results of the mediating of public low-carbon behavior and medical burden of residential households.

Variable	Public low-carbon behavior			Medical burden of residential households		
	*LC_beh*	*Re_phex*	*Ab-phex*	*MB_rh*	*Re_phex*	*Ab-phex*
*DID*	0.081** (0.03)	−0.321*** (0.08)	0.435*** (0.15)	−0.218*** (0.07)	−0.326*** (0.07)	0.350** (0.14)
*LC_beh*	/	−0.119** (0.05)	−0.318*** (0.09)	/	/	/
*MB_rh*	/	/	/	/	0.023 (0.02)	−0.271*** (0.03)
Control variables	Yes	Yes	Yes	Yes	Yes	Yes
Urban fixed effects	Yes	Yes	Yes	Yes	Yes	Yes
Time fixed effects	Yes	Yes	Yes	Yes	Yes	Yes
*Cons*	6.080*** (0.36)	2.936*** (0.79)	45.556*** (1.523)	5.186*** (0.75)	2.087** (0.83)	45.026*** (1.35)
*Bootstrap test 95%CI*	/	[0.030, 0.066]	[0.135, 0.292]	/	[0.001, 0.020]	[0.050, 0.289]
*N*	4,836	4,836	4,836	4,836	4,836	4,836
*R-squared*	0.717	0.888	0.977	0.430	0.887	0.977

Standard Error in parentheses, ***, **, and * mean passing significance test at 1, 5, and 10% confidence levels, respectively.

#### 4.4.2 Testing the mediating mechanism of the medical burden on residential households

Table 7 presents the regression results with the medical burden on residential households as the mediating variable. The regression coefficient of *DID* for the medical burden on residential households is −0.218 and is significant at the 1% level. This indicates that the LCCP policy has significantly improved the health level of residents and reduced the medical burden on residential households. Once the mediating variable is included, the coefficient of *DID* for relative public health expenditure is −0.326 and is significant at the 1% level, while the coefficient of the medical burden on residential households is 0.023, but is not significant. This shows that the medical burden on residential households does not play a mediating role in the relationship between *DID* and absolute public health expenditure. Once the mediating variable is included, the coefficient of *DID* for absolute public health expenditure is 0.350 and is significant at the 5% level, while the coefficient of the medical burden on residential households is −0.271 and is significant at the 1% level. This indicates that the LCCP policy has significantly reduced both the medical burden on residential households and absolute public health expenditure, and that there is a mediating effect. Hypothesis 3 is thus supported.

Bootstrap tests were also conducted on the mediating roles of the public’s low-carbon behaviors and the medical burden on residential households. As can be seen from the test results in Table 7, the confidence intervals of the Bootstrap tests on the public’s low-carbon behaviors and the medical burden on residential households for the two explanatory variables do not include 0, thus confirming the robustness of the results on the mediating effects.

## 5 Discussion

Globally, the interaction of low-carbon policies with public health expenditures has become a core issue in environment-health collaborative governance. The EU Carbon Border Adjustment Mechanism (CBAM) enhances regional emissions reduction effects by mitigating carbon leakage risks, with its potential health co-benefits manifested as a reduced disease burden associated with air pollution (45). The Paris Climate Change Agreement emphasizes the necessity of incorporating public health considerations into climate policies. The practices of Switzerland, Iran, Germany, and Spain demonstrate that incorporating health indicators into pollution control strategies yields significant public health benefits (46–49). Nordic countries such as Sweden and Norway use carbon revenue to fund health programs promoting sustainable and healthy lifestyles, resulting in a heightened sense of health benefits among their citizens (50). Carbon pricing has generated substantial health co-benefits for Canada through environmental improvements and public engagement in low-carbon initiatives (51). Developing nations like Indonesia and India have successfully reduced waste-related emissions using command-and-control policies that enable urban areas to enhance their air quality and mitigate pollution-induced health risks (52, 53). For countries with weaker fiscal capacities, the “carbon tax + earmarked health transfer payments” model is a way to prevent low-carbon investments from crowding out essential health expenditures (54). These cross-national experiences collectively demonstrate that embedding low-carbon policies within a health-oriented design can overcome the cost dilemma in environmental governance to achieve the simultaneous enhancement of ecological benefits and public welfare. However, current international practices have yet to adequately address two critical propositions. First, under the dual constraints of rapid industrialization and centralized governance, how can the risk of low-carbon investments crowding out public health resources best be resolved? Second, in economies with highly uneven regional development, how can differentiated policy instruments best be used to reconcile environmental regulation with health equity? As the largest developing nation in the world, China’s LCCP policy represents a unique operational pathway that offers a comparative framework for other nations. This study focuses on the Chinese experience, seeking to unravel the mechanism through which low-carbon policies interact with public health, thereby providing differentiated reference models to provide guidance for other economies.

### 5.1 The inherent contradiction and the interactive effect of China’s LCCP policy on public health expenditure

The results of this study show that the LCCP policy has an asymmetric effect on public health expenditure. Specifically, the LCCP policy has a significantly negative impact on the relative amount of public health expenditure, but a significantly positive impact on the absolute amount of public health expenditure. The independent effects and interaction mechanisms by which the LCCP policy affects these two aspects of public health expenditure are as follows:

The pilot low-carbon cities had a noticeable effect of reducing relative public health expenditure, consistent with the findings of most studies to date (55). China’s LCCP policy has achieved remarkable results. Under the premise of reducing the input of ecological resources and output of pollutant emissions, these policies have improved the overall level of public health. Low-carbon city construction is a process of the coordinated development of the economy-environment-health system. Low-carbon cities positively affect residents’ health through the three aspects of economy, environment, and lifestyle. Also, the improvement of peoples’ health level objectively saves the public health financial supply of the Chinese government (56). Conversely, the decentralization of China’s fiscal system has resulted in substantial local financial autonomy, substantially influencing two critical areas of public welfare: healthcare and environmental quality. Since 2009, China’s performance evaluation system for officials increasingly emphasizes the assessment of energy consumption and carbon emissions. Since 2012, there has been a pronounced transition in the competitive strategies of prefecture-level city governments, which shifted from a focus on “competition for growth” to an emphasis on “green and low-carbon development” (57). Consequently, it can be inferred that governments in pilot regions of the LCCP policy may prioritize expenditures on low-carbon development initiatives, thereby potentially delegating greater responsibility for the provision of healthcare services to market mechanisms.

The LCCP policy has had a significant promoting effect on absolute public health expenditure. There may be a policy synergy and linkage effect as well as an economic growth mechanism underlying the continuous expansion of the scale of absolute public health expenditure in the LCCP pilot cities. Environmental and ecological protection, along with public health services, are people-centered public services, administered under governmental leadership. Substantial theoretical and policy-based compatibility exists between these two domains. China’s 14th Five-Year Plan prioritizes the enhancement of citizens’ wellbeing. A comprehensive set of Healthy China policy documents has been released to delineate the strategic direction and timeline for the development of the public health service system at the macro level. This initiative has led to the establishment of a policy framework that encourages local governments to actively engage in national competition to improve public health services. Consequently, the effectiveness of public health initiatives has become a key component of the government’s performance evaluation system (58). Secondly, the LCCP policy can influence public health expenditure levels via mechanisms of economic growth. Low-carbon policies compel enterprises to modernize both their production techniques and capacity structures, thereby achieving intensive growth by enhancing green competitiveness and mitigating adverse environmental externalities (59). Conversely, pilot cities have adopted green fiscal policies and stimulated the green financial market through the issuance of green bonds and green credits; however, they have not yet fully realized the dual benefits of invigorating economic vitality and enhancing environmental governance (34). The economic development level of a certain region considerably influences its local financial capacity. Consequently, enhancements in economic development, as stimulated by the LCCP policy, can substantially bolster the efficiency of public health expenditure.

In conclusion, the inherent contradiction between a relative decrease vs. an absolute increase in public health expenditure under the LCCP framework essentially reflects a conflict of goals between environmental protection priorities and livelihood security. Although the core objectives of low-carbon policies lie in carbon emissions reduction and environmental quality improvement, their implementation requires fiscal resource reallocation that may lead to a relative deprioritization of public health in local government expenditure planning. Conversely, as a fundamental requirement of livelihood, public health necessitates a steady or growing absolute expenditure, thus creating an inherent tension in policy objectives. Public health and environmental regulation are not mutually independent domains; over time they progressively move toward expenditure-carbon target coupling. Our findings validate this dynamic: the low-carbon city pilot policy demonstrates transient suppression effects on relative public health expenditure but exhibits long-term persistence in elevating absolute expenditure levels. This manifests the two-sided nature of resource allocation between short-term crowding-out and long-term synergy. In the short term, environmental investments crowd out other budgetary allocations causing relative expenditure decline. But in the long term, environmental quality improvements reduce disease burdens, lower healthcare costs, and release fiscal space that can reciprocally reinforce public health investments.

### 5.2 Heterogeneity of the impact of the LCCP policy on public health expenditure: regions, medical infrastructure, and pollution levels

The impact of the LCCP policy on public health expenditure demonstrates significant regional disparities, fundamentally reflecting differences in developmental stages and resource endowments. The LCCP policy had pronounced inhibitory effects on relative public health expenditure in the eastern region, but limited influence on absolute expenditure levels. Eastern China’s substantial economic scale advantages enable more intensive implementation of the LCCP policy, as manifested in technological substitution and efficiency gains driving low-carbon transition, environmental improvements reducing therapeutic expenditures, and optimization of expenditure structure (60, 61). The region’s rigid health demands constrain total expenditure growth, while high fiscal self-sufficiency elasticity enables the stabilization of overall health spending (41). In the central region, the LCCP policy had negligible effects in reducing relative public health expenditure but significantly enhanced absolute expenditure levels. As this area is less developed, central China’s low-carbon development prioritizes the enhancement of regional competitiveness under carbon constraints (62). Industrial transformation and policy dividends increase the scale of health expenditure, although legacy pollution-induced disease burdens from industrial cities dilute their relative effectiveness. In the western region, the LCCP policy had minimal impact on relative public health expenditure but reduced the absolute amount of expenditure. In resource-dependent economies, high-cost low-carbon transitions tend to crowd out other budgetary allocations, temporarily attenuating relative effects (63). The weak efficacy of environmental regulations in western areas renders limited fiscal resources vulnerable to crowding-out by low-carbon investments, resulting in the passive compression of health expenditures (64).

The impact of the LCCP policy on public expenditure demonstrates a profound dependence on local healthcare infrastructure, mediated by resource redundancy and health service elasticity. In regions with abundant medical resources, the LCCP policy had negligible effects on the relative amount of public health expenditure, whereas areas with average medical resources experienced significant reductions in the relative amount of expenditure. Resource-rich healthcare systems likely have stabilized expenditure structures with stronger adaptability to the LCCP policy and limited expenditure adjustment flexibility (65). Moreover, high-density talent pools and institutional frameworks create fixed costs that are resistant to rapid expenditure restructuring through policy interventions. Conversely, regions with moderate medical resources demonstrated a heightened sensitivity to LCCP policy-induced health demand benefits. Pollution control rapidly reduces environment-related disease burdens, directly compressing the proportion of therapeutic expenditures. Notably, the LCCP policy’s enhancement of absolute public health expenditure proved to be more substantial in areas with abundant medical resources, compared to more average regions. This disparity likely stems from scale effects that dilute intervention costs. Empirical studies have revealed statistically significant diminishing marginal returns on government health resource inputs (66). Our empirical analysis corroborates this pattern: average-resource regions operated along steeper segments of health production curves, while resource-abundant areas functioned within relatively flat intervals.

The impact of the LCCP policy on public expenditure also exhibited environmental pollution gradient differentials. Our results reveal that the policy reduced absolute public health expenditure in high-pollution zones, but increased it in low-pollution areas. This confirms the LCCP policy’s ability to suppress pollution-induced health damage and the existence of marginal cost differentials (67). In medium-high pollution regions that must rely on mandatory emission reductions, significant health benefits facilitate policy effectiveness, easily forming a virtuous cycle of “policy-driven emission reduction → health expenditure decline → fiscal resource liberation → further emission reduction.” Conversely, low-pollution zones require higher technological inputs for additional emission cuts, and thus increasing marginal costs constrain policy efficacy, manifesting as an increase in expenditures (68). The LCCP policy’s inhibitory effects on relative public health expenditure were highest in low-pollution areas, followed by high-pollution areas, with medium-pollution zones experiencing the least effect. Lower environmental health risks in low-pollution areas enable policy-driven expenditure shifting from treatment to prevention, with preventive services constituting a lower proportion of overall cost. In medium-high pollution regions, short-term transitional costs from environmental remediation necessitate the continued management of legacy pollution health impacts, partially offsetting the LCCP policy’s expenditure reduction effects.

### 5.3 The public constraint effect of the LCCP policy: the mediating effect of public low-carbon behaviors

Public low-carbon behaviors play a negative mediating role in the impact of the LCCP policy on both absolute and relative public health expenditure, confirming the environmental health spillover effect of the LCCP. This indicates that the low-carbon behaviors of the public force reductions in public health expenditure by reducing medical needs and reshaping fiscal priorities. There are several possible reasons for this. First, a low-carbon lifestyle has a good health-benefit ratio. Implementing the LCCP policy can disrupt entrenched patterns of residential carbon emissions. These policies facilitate the transition of people toward sustainable lifestyles by fostering awareness of environmentally friendly practices and values (69). Furthermore, promoting green and low-carbon behaviors among the public can substantially decrease both carbon emissions and indoor pollutant levels, thereby enhancing public health and consequently lowering healthcare expenditures (33). Research has indicated that public health outcomes improve substantially when more than 90% of the population engages in environmental governance (70). Furthermore, individual low-carbon behaviors should be grounded in moral values, a sense of responsibility, and a preference for environmentally sustainable practices. Integrating public participatory environmental regulation into the core of current practices through social construction, the development of specific implementation pathways, and the enhancement of incentives for sustainable living is essential. To make the health value explicit, it is necessary to promote the transformation of the public perception of low-carbon behaviors from an issue of environmental protection obligation to one of health rights. Second, the transformation of the economic dividends of low-carbon behaviors is relevant to this effect. The adoption of low-carbon lifestyles by the public facilitates the advancement of low-carbon economic development, thereby enhancing environmental welfare outcomes associated with LCCP policies. Environmental awareness serves as a critical precursor to green purchasing behavior, exerting a significant influence on consumers’ green and low-carbon consumption patterns; this influence intensifies over time (71). According to the “green consumption – low carbon production” complementary model, the establishment of the low-carbon consumption concept can be expected to influence consumer demand preferences toward environmentally friendly options. This shift will likely increase the propensity of the public to purchase low-carbon products, thereby facilitating the green upgrading of the demand structure. Consequently, this transition also encourages enterprises to shift their production focus from conventional products to low-carbon alternatives. Furthermore, the initiatives and efforts of businesses related to low-carbon practices can be anticipated to generate positive spillover effects, ultimately decreasing governmental expenditures on environmental protection and mitigating the loss of environmental health. Third, the self-reinforcement of the prioritization of low-carbon policies plays a role. Enhanced public low-carbon behaviors improve environmental governance performance, thereby intensifying local governments’ performance incentive dependency on low-carbon initiatives. Consequently, pilot city governments’ fiscal increments prioritize environmental incentives and green infrastructure investments, leading to a relative dilution of public health expenditure as a portion of the whole.

### 5.4 The health effects of the LCCP policy: the mediating effect of the medical burden on residential households

The medical burden on residential households played a significant positive mediating role in the LCCP policy’s impact on absolute public health expenditure, but played a non-significant negative mediating role in its effect on relative expenditure. The notable increase in absolute health expenditure likely stems from two mechanisms. First, low-carbon policies effectively reduce pollution-related disease incidence through air quality improvement and living environment optimization (23, 37, 72), directly alleviating the pressure of household medical expenditures. Second, reduced medical burdens may free up household consumption capacity, stimulating domestic demand that can boost local economic growth, thereby creating fiscal space for governments to expand public health expenditure. This virtuous cycle of “environmental governance→health improvement→economic development” exemplifies the synergistic health benefits of low-carbon policies. However, the non-significant negative mediating effect of household medical burden in the LCCP policy’s effect on relative public health expenditure exposes latent dilemmas of policy coordination. This may be closely related to local governments’ fiscal rebalancing mechanisms. Specifically, when sustained fiscal commitments to low-carbon urban development are required, local governments may adjust their expenditure structures through two pathways: either directly cutting health budgets to fund low-carbon projects, or misjudging actual medical needs due to the crowding-out effect from increased household medical expenditures that consequently reduces public investments. These differing mediation effects reveal the hierarchical transmission characteristics of policy impacts. There are two potential explanations for this. (1) The environmental health benefits of low-carbon policies are temporally lagged, while medical burden increases induced by economic restructuring are immediate. Structural unemployment during traditional industry transitions reduces household incomes in regions with underdeveloped social security systems, forcing higher out-of-pocket medical expenditure ratios (73). (2) Current policy designs lack medical compensation mechanisms that can effectively offset transitional social costs. Enhanced environmental regulations increase operational costs for SMEs, prompting reductions in benefits including employee health insurance, thereby indirectly transferring medical costs to households (74). These findings agree with Francesco’s (2023) conclusions on the distributional effects of climate policy, suggesting a need for refined policy coordination designs (75).

### 5.5 Limitations and research outlook

Despite the important findings of this study, several limitations should be acknowledged: First, this research is centered on the LCCP policy of China, a developing nation, which may limit the generalizability of the conclusions to other countries or regions. This limitation arises from variations in economic development levels, ideologies, and environmental regulations between developing and developed countries. Second, because of the lack of municipal-level indicators, the evaluation of public health expenditure relies entirely on proxy indicators at the provincial level. The underlying assumption posits that the LCCP policy will influence public health expenditure at both the local and provincial levels through spatial spillover effects. While this approach has been supported by a substantial body of literature and possesses a degree of rationality, the potential for measurement bias persists, which may compromise the accuracy of the obtained results. Third, this study lacks an analysis of public perception. Due to the aggregate-level administrative data used in this research, we were unable to assess how individual citizens perceive the trade-offs between environmental policies and healthcare resource allocation. Furthermore, our focus on expenditure magnitudes does not capture distributional equity in healthcare access across regions.

Future research can be optimized and expanded in the following ways: Firstly, efforts should be directed toward developing a low-carbon policy framework from a global perspective. This involves conducting comparative analyses of the impacts environmental regulations have on public health across different countries; such analyses would facilitate the generation of more universal and comprehensive research conclusions. Secondly, it is essential to identify and utilize direct evaluation indicators to assess public health expenditures in the LCCP policy cities. Thirdly, mixed-method studies combining econometric analysis with public opinion surveys could be conducted, applying distributive justice frameworks to environmental-health policy evaluations.

## 6 Conclusion

This paper treats China’s LCCP policy as a natural experiment and assesses its effects on public health expenditures by integrating macro-level and micro-level data. Our results show that the LCCP policy significantly reduced the relative proportion of public health expenditure, but at the same time expanded its absolute scale. The heterogeneity analysis shows that differences in resource endowments lead to significant regional differences in the impact of the LCCP policy on public health expenditure. Due to resource redundancy and the elasticity of health services, areas with abundant medical resources may have already formed a stable structure of health expenditure, while areas with merely average medical resources are sensitive to the LCCP policy’s health benefit demands. High and moderately polluted areas rely on mandatory emissions reduction, and the health benefits are significantly higher there than in low-pollution areas. Analysis of the underlying mechanism indicates that the LCCP policy can encourage the public to develop good habits of low-carbon behavior, which in turn significantly and negatively affect the scale and proportion of public health expenditure. The low-carbon city pilot policy can also alleviate the medical burden on residents’ households, thereby reducing the absolute amount of public health expenditure. It is important to highlight that environmental regulatory policies and public health services exert a “qualitative” impact as opposed to a “quantitative” impact. Policy design has regional characteristics, but the conclusions of this study can provide a theoretical reference for the coordination of international climate policies. The policy implications and suggestions are as follows.

1. The asymmetric effects of the LCCP policy on public health expenditure fundamentally exemplify the goal conflict between environmental prioritization and livelihood protection, necessitating the concurrent advancement of environmental governance and the equitable allocation of healthcare resources. We recommend establishing synergistic budgeting mechanisms that operationalize the nexus of environmental regulations and health benefits through fiscal conversion instruments. This should involve setting GDP proportion thresholds for health expenditures so that low-carbon institutional arrangements and healthcare budgets can be dynamically adjusted to ensure that public health spending growth rates outpace the economic growth induced by low-carbon transitions.
2. The multidimensional heterogeneity in the impacts of the LCCP policy on public health expenditures reveals critical implementation insights. Specifically: regional disparities reflect the compatibility of economic foundations with policy instruments; healthcare resource differentials determine expenditure restructuring capacity; and pollution gradients mirror marginal health benefit variations in environmental remediation. Future policies should adopt precision governance through cost-effective technological solutions, regional collaboration (i.e., technology sharing and ecological compensation), and institutional innovation to achieve dual optimization of decarbonization and the advancement of public health.
3. The mediating effects of public low-carbon behaviors reveal intrinsic linkages of environment and health governance. Strategic recommendations include intensifying low-carbon behavioral guidance through community education and carbon credit reward systems, thereby directly correlating pro-environmental actions (i.e., green commuting, waste sorting) with personal health benefits. This would cultivate a self-reinforcing virtuous cycle of low carbon and health, transforming individual choices into enhancements of the collective welfare.
4. The mediation pathway through household medical burdens highlights the health co-benefits of low-carbon policies. Policy prescriptions include: instituting environmental health impact assessment frameworks that incorporate hidden costs such as medical burdens; creating cross-departmental health-environment collaborative governance mechanisms to strengthen policy synergies; and establishing transitional compensation funds to mitigate restructuring costs for vulnerable groups, thereby ensuring the equitable distribution of the benefits of low-carbon transition.

## Data Availability

The original contributions presented in the study are included in the article/supplementary material, further inquiries can be directed to the corresponding author.
